# *Rhopalurus junceus* scorpion venom induces G2/M cell cycle arrest and apoptotic cell death in human non-small lung cancer cell lines

**DOI:** 10.1590/1678-9199-JVATITD-2024-0035

**Published:** 2025-02-03

**Authors:** Alexis Díaz-García, Ángel Garrido, Jenny Laura Ruiz-Fuentes, Tamara Hermosilla, Diego Varela

**Affiliations:** 1 LifEscozul Chile, Santiago, Chile.; 2 Millennium Nucleus of Ion Channel-Associated Diseases (MiNICAD), University of Chile, Santiago, Chile.; 3 Physiology and Biophysics Program, Institute of Biomedical Sciences, Faculty of Medicine, University of Chile, Santiago, Chile.

**Keywords:** Apoptosis, Cell cycle arrest, Rhopalurus junceus, Scorpion venom, Synergism

## Abstract

**Background:**

Non-small cell lung cancers (NSCLC) represent the primary cause of cancer-related deaths worldwide. *Rhopalurus junceus* venom has been shown to exert cytotoxic effects against a panel of epithelial cancer cells *in vitro* and suggested that NSCLC was the subtype most susceptible to the treatment.

**Methods:**

This study evaluated the effect of *Rhopalurus junceus* scorpion venom on cell viability, in non-cancerous (MRC-5, lung; CHO-K1, ovary) and NSCLC (A549; NCI-H460) cell lines. The effects on cell cycle, apoptosis, and cell signaling-related proteins were determined by flow cytometry and WB. Protein fractions responsible for the observed effect were identified using HPLC.

**Results:**

Scorpion venom was more effective against NSCLC than non-cancerous cells. E_max_ values were 20.0 ± 5.8% and 22.47 ± 6.02% in A549 and NCI-H460 cancer cells, respectively, as compared to 50 ± 8.1% in MRC-5 and 54.99 ± 7.39% in CHO-K1 cells. It arrested NSCLC cells in the G2/M phase, while non-cancerous cells were arrested in the S (MRC-5) or G0/G1 (CHO-K1) phases. No changes were observed in the Bax/Bcl-2 or the cleaved-caspase 3/Total caspase 3 ratios in cells treated with venom. Likewise, the scorpion venom treatment did not affect p-ERK, p-AKT, or p-38MAPK protein levels. In contrast, scorpion venom treatment increased the cytosolic apoptosis-inducing factor (AIF) in A549 cells, indicating caspase-independent apoptosis. Additionally, combined etoposide/venom exposure provoked G2/M arrest and apoptosis in NSCLC more strongly than either substance alone. Furthermore, upon crude venom fractioning through RP-HPLC, we found two soluble fractions with high cytotoxic effects.

**Conclusion:**

The present study concludes that a specific fraction of *Rhopalurus junceus* venom reduces cell viability of NSCLC cells. The AIF protein plays a key role in mediating caspase-independent apoptotic cell death. These findings suggest that *Rhopalurus junceus* venom enhances the anticancer effect of etoposide *in vitro* by causing cell cycle arrest and caspase-independent apoptosis.

## Background

Lung cancers are the second-most aggressive and common malignancy worldwide, with an estimated 2.2 million new cases and 1.8 million deaths per year. This cancer is responsible for 18% of all malignancies [1]. Approximately 85% of total pulmonary cancer diagnoses belong to one of the non-small cell lung cancers (NSCLC) histological subtypes [1, 2]. The 5-year survival rate for NSCLC is only 15% due to frequent recurrence and progression after surgery and treatment with chemo- and radiation therapies [1, 2]. This highlights the critical need for new treatment strategies against NSCLC [3-5]. 

Scorpion venoms have been tested as anticancer agents against epithelial cancer cells [6-11]. Promising results suggest that these arthropods are a natural source of new compounds for NSCLC therapy. The mechanisms underlying the anticancer properties of scorpion venoms seem to involve cell cycle arrest, leading to apoptotic cancer cell death [7, 8, 10].

Scorpion venom is a rich, highly complex, and heterogeneous source of biomolecules, including small-molecule peptides and enzymes such as hyaluronidase and phospholipase. Peptides are the most studied components due to their key roles in the toxicological and pharmacological effects of scorpion venoms [12]. 

*Rhopalurus junceus* (*R. junceus*) is one of Cuba’s most widespread endemic scorpion species, and its venom has long been used in popular Cuban traditional medicine. *R. junceus* venom has been shown to exert cytotoxic effects against epithelial cancer cells *in vitro* and inhibit cancer progression in a murine breast cancer model [13, 14]. Interestingly, an *in vitro* evaluation of *R. junceus* against a panel of cancer cell lines suggested that NSCLC was the subtype most susceptible to the treatment [13]. Preliminary results analyzing mRNA expression in the human breast cancer cell line MDA-MB-231 suggest that apoptosis may be the preferred mechanism of cell death for this natural extract [15] in general. However, the process underlying cancer cell death after *R. junceus* treatment is yet to be investigated in NSCLC.

Cell proliferation is based on the progression of the cell cycle through four phases: gap 1 (G1), DNA synthesis (S), gap 2 (G2), and mitosis (M) [16]. Due to dysregulation or loss of checkpoint integrity, cancer cells typically fail to stop at the normal cell cycle checkpoints, leading to uncontrolled proliferation [17, 18]. Thus, arresting the cells at some point in the cycle is sometimes a successful therapeutic strategy. In these cases, sustained cell cycle arrest due to the severity of DNA damage leads to an irreversible exit from the cycle, causing cancer cell death [16-18].

Certain types of anticancer treatments, such as topotecan [19], doxorubicin [20], vincristine [21], or etoposide [22], induce cell cycle arrest due to DNA damage. This damage impairs cell cycle progression across G1, S, or G2/M checkpoints, ultimately preventing the completion of mitosis. However, some therapeutic properties are usually limited and accompanied by unwanted collateral effects. Etoposide, a semi-synthetic derivative of podophyllotoxin, is usually employed in NSCLC treatment. This agent, inhibits topoisomerase II leading to the formation of single- and double-strand DNA breaks [22], inducing cell cycle arrest at S and G2/M phases, followed by apoptotic cell death. However, like other chemotherapeutic strategies, the therapeutic potential of etoposide-based treatment is limited by serious side effects and resistance [22, 23]. Therefore, it is crucial to research new combinations of drugs that might enhance the efficacy of etoposide and other conventional chemotherapeutics. *R. junceus* venom is a promising natural therapeutic strategy emerging as an anticancer treatment.

In this work, we evaluate the effects of *R. junceus* venom on cell viability in non-cancerous and cancerous NSCLC cell lines using cell viability assays, flow cytometry, and Western blot. Moreover, we assess the combined effects of venom and etoposide on cell cycle phase distribution and cancer cell death mechanisms.

## Methods

### Reagents 

Dulbecco’s modified Eagle’s medium was purchased from GIBCO/BRL (Gaithersburg, MD). Fetal bovine serum (FBS) was purchased from Biological Industries. The 3-[4,5-dimethylth-iazol-2-yl]-2,5-diphenyl tetrazolium bromide (MTT) reagents were obtained from Merck (Merck, USA). Propidium iodide, RNase A, and Micro BCA Protein Assay Kit were obtained from Thermo Fisher. Annexin V FITC Early Apoptosis Detection Assay Kit was obtained from Cell Signaling Technology. Etoposide was obtained from Cayman Chemical.

### Venom source

Venom was obtained from LifEscozul company. Briefly, scorpion venom was obtained from adult *R. junceus* scorpions by electrical stimulation, centrifuged at 13,000 rpm for 15 min, and soluble protein supernatant filtered through a 0.2-µm syringe filter. Scorpion venom protein content was determined using the Micro BCA Protein Assay Kit**.** The protein concentration of the scorpion venom was 5.3 mg/mL and was used to prepare the venom dilutions tested against the cell lines.

### Cell lines

The NSCLC A549 (CCL-185™) and NCI-H460 (HTB-177™) cell lines were obtained from ATCC. Non-cancerous MRC-5 (T0016002) and CHO-K1 (P0017001) cells were obtained from AddexBio. All cells were maintained in Dulbecco's modified Eagle's medium, 90% (w/v) with heat-inactivated fetal bovine serum (FBS), 10% (v/v), penicillin (100 U/mL), streptomycin (100 μg/mL), and fungizone (100 μg/mL).

### Cell viability (MTT assay)

Cell viability was assessed using MTT assay [24]. The cell lines were seeded into a 96-well plate at a density of 5 × 10^3^ cells/well, and various scorpion venom concentrations were added (0, 0.125, 0.25, 0.5, 0.75, 1, 1.5, 2 mg/mL). The 96-well plate was incubated for 72 h at 37°C, 5% CO_2_. Then, 10 µL MTT solution (5 mg/mL) (Merck, USA) was added to the 96-well culture plates, and cells were incubated for 3 h at 37°C, 5% CO_2_. The culture medium was decanted, and 150 µL DMSO (100 %) was added to each well. A_560_ nm values were obtained with the Synergy™ HTX Multi-Mode Microplate Reader (Agilent BioTek, USA). E_max_ and before IC_50_ values were obtained from concentration-effect curves as follows: 

Experiments were repeated three times, and five technical replicates were used. The culture medium was used as the negative control.

### Cell cycle analysis 

Each cell line was cultured in 60-mm dishes at a density of 5 × 10^5^ cells/plate. After incubation at 37°C, 5% CO_2_ overnight, cells were treated with the corresponding ½IC_50_, IC_50_, and 2xIC_50_ doses for 48 h. The 48-h incubation period was chosen to minimize the confounding effects of primary cell death, especially at the highest venom doses. Cells were harvested by trypsinization and washed twice with ice-cold phosphate-buffered saline (PBS, pH 7.4). For the cell cycle analysis, cells were fixed in ice-cold methanol (100%) for 1 h at -20°C. Cells were washed twice with ice-cold PBS, centrifuged at 1500 rpm for 5 min at 4°C, re-suspended in ice-cold PBS with 100 µg/mL RNase A (Thermo Scientific, USA), and incubated at 37°C for 1 h. Finally, 50 µg/mL propidium iodide solution (Merck, USA) was added, and cells incubated at room temperature for 15 min in darkness. Cell cycle phase was determined by flow cytometry and analyzed with FlowJo software. For each experiment, 10,000 events were recorded, assays were carried out in duplicate, and experiments were repeated three times.

### Cell synchronization

A metabolic strategy was used to perform cell synchronization. For the cell cycle synchronization at the G0/G1 phase, A549 cells were seeded in 60-mm dishes at 5×10^5^ cells/plate density and incubated at 37°C, 5% CO_2_ overnight. The culture medium was discarded, and the cells were washed twice with PBS. Cells were exposed to fetal bovine serum-free culture medium for 3 days. Finally, cells were washed twice with PBS and separated into four groups. The first group of cells was washed twice with PBS and processed for flow cytometry. This group served as the cell synchronization control. In the second group, cells were washed twice with PBS and released into a complete culture medium for 48 h. The third group of cells was washed twice with PBS and treated with 2xIC_50_ for 24 h. In the fourth group, cells were washed twice with PBS and treated with 2xIC_50_ for 48 h.

### Cell death determination 

The apoptosis event was identified by flow cytometry with the Annexin V-FITC/PI kit (Cell Signaling Technologies, USA). Cancer cells were seeded in 60-mm dishes (5 × 10^5^ cells/well) and cultured at 37°C, 5% CO_2_ overnight. After this period, cells were treated with ½IC_50_, IC_50,_ and 2xIC_50_ doses of scorpion venom and incubated at 37°C, 5% CO_2_, for 48 h. Each treatment group was individually harvested by trypsinization, washed twice with ice-cold PBS, and centrifuged at 1500 rpm for 5 min at 4°C. The pellets were suspended in a binding buffer (250 μL) and stained with Annexin V-FITC/PI following the manufacturer’s recommendations. Cells were incubated for 10 min at 4°C in the dark, and apoptosis was detected by flow cytometry and analyzed with FlowJo software. For each experiment, 10 000 events were recorded, assays were carried out in duplicate, and experiments were repeated three times.

Apoptotic cell death was analyzed in serum-starved synchronized cells. After 72 h of serum starvation, cells were washed twice with PBS and separated into four groups. The first group of cells was washed twice with PBS; this group served as the control. In the second group, cells were washed twice with PBS and released into a complete culture medium for 48 h. The third group of cells were washed twice with PBS and treated with 2xIC_50_ for 24 h, and in the fourth group, cells were washed twice with PBS and treated with 2xIC_50_ for 48 h. All groups were individually harvested by trypsinization, washed twice with ice-cold PBS, centrifuged at 1500 rpm for 5 min at 4°C, and assayed for apoptotic cell death as above, following the manufacturer’s recommendations.

### Combined etoposide plus R. junceus scorpion venom treatment

The cytotoxicity of etoposide alone and combined with scorpion venom was evaluated through the MTT assay. A549 cells were seeded in a 96-well microplate at 5 × 10^3^ cells/well and incubated overnight at 37°C, 5% CO_2_. The IC_50_ value for etoposide was obtained from the concentration-response curve, with concentrations ranging from 0-20 µM. For the combined treatment analysis, cells were treated with scorpion venom concentrations (½IC_50_, IC_50_, 2xIC_50_) combined with etoposide (IC_50_) for 72 h at 37°C, 5% CO_2_. The culture medium was used as the negative control. The drug interaction analysis was performed using the combination index (CI). The CI was generated automatically using CompuSyn software (version 1.0; ComboSyn, Inc., Paramus, NJ, USA), as previously described by Chou [25]. CI < 1 indicates synergism; CI = 1 or close to 1 indicates additive effects, and CI > 1 indicates antagonism. Cancer cell cycle and cell death results for IC_50_ doses of scorpion venom alone, etoposide alone, and combined venom/etoposide were determined through flow cytometry as described above.

### Western blot analysis

Cell proteins were extracted from the cell pellets of A549 cells using RIPA lysis buffer (25 mM Tris-HCl, pH 7.6, 150 mM NaCl, 1 mM EDTA, 1% Triton, 0.1% SDS, sodium deoxycholate) for 30 min. The proteins were separated by denaturing sodium dodecyl sulfate polyacrylamide gel electrophoresis (SDS-PAGE; 10-15%) then transferred onto a nitrocellulose blotting membrane (Amersham, Cytiva, Germany). The membrane was blocked with 5% non-fat milk for 1 h, followed by overnight incubation at 4°C with the primary antibodies against p44/42 MAPK (Erk1/2) [Cell Signaling Technology (CST), Cat. No. 9102, 1:1000], phospho-p44/42 MAPK (Erk1/2) (Thr202/Tyr204) (CST, Cat. No. 9101, 1:1000), caspase-3 (D3R6Y) (CST, Cat. No. 14220, 1:1000), alpha tubulin (DM1A) (Novus Biologicals, Cat. No. nb100-690, 1:5000), Bcl-2 (114) (CST, Cat. No. 15071, 1:1000), Bax (CST, Cat. No. 2772, 1:1000), p38 MAPK (CST, Cat. No. 9212, 1:1000), phospho-p38 MAPK (Thr180/Tyr182) (CST, Cat. No. 9211, 1:1000), Akt (pan) (C67E7) (CST, Cat. No. 4691, 1:1000), phospho-Akt (Ser473) (D9E) XP (CST, Cat. No. 4060, 1:2000) and anti-AIF (1:1000, sc-13116; Santa Cruz Biotechnology Inc., Santa Cruz, CA, USA). Following incubation with peroxidase-conjugated goat anti-rabbit IgG at room temperature for 1 h, proteins were visualized using the SuperSignal West Pico PLUS Chemiluminescent substrate kit (Thermo Scientific, USA) and detected using a chemiluminescence analyzer. All signals from proteins of interest were normalized based on the (-tubulin or (-actin signals. WB raw data are included as supplementary material (Additional files 1 to 3).

### Scorpion venom fractionation by reversed-phase chromatography

The R. junceus scorpion venom was analyzed by reversed-phase high-performance liquid chromatography (HPLC) using a C18-phase reverse column (Intersustain C18, pore size 5 µm, column size 4.6 mm I.D. x 250 mm., GL Science Inc. Japan). The mobile phases were as follows: phase A - 0.1% TFA in HPLC grade water; phase B - 0,1% TFA in acetonitrile. The fractionation procedure was performed by injecting 6 mg of the venom dissolved in water and was eluted at 1 mL/min flow rate using a linear gradient of 0-45% of solvent B for 45 min, then increased linearly to 100% solvent. The column temperature was set at 26°C, and the UV absorbance was monitored at 230 nm. Various runs were performed, and fractions were collected and evaluated for cell viability effect against A549 cancer cells.

The chromatographic profile at 3-28 min retention times was subdivided into five fractions collected individually. All collected fractions with similar retention times were pooled and dried overnight at 4°C in a concentrator (Centrivap concentrator system, Labconco, United States). Finally, the concentrated fractions were stored at -20°C until use.

A549 cancer cells and MRC-5 were treated individually with each fraction at 0.5 mg/mL for 72 h at 37°C, 5% CO_2_, and the effect was observed through the MTT assay as described above. Afterward, different concentrations from the most active fractions were applied (15.1, 31.25, 62.5, 125, 250, 500, 1000 µg/mL) to A549 cells and treated for 72 h at 37°C, 5% CO_2_ and the effect was observed through the MTT assay. Finally, 150 µL DMSO was added to each well to dissolve the formazan crystals and the absorbance was detected at 560 nm. IC_50_ values were determined as above. The experiments were performed three times with three technical replicates each time.

### Statistical analysis 

The IC_50_ values for scorpion venom and fractions were determined using non-linear regression curves. Normality tests were carried out for all data. ANOVA or Kruskal-Wallis tests were used for multigroup comparisons of IC_50_ and E_max_ values, as well as cell cycle distribution, cell death events, and combined treatment results. The Mann-Whitney U test was used for two-group comparisons. GraphPad Prism version 5.01 for Windows, (GraphPad Software, San Diego California, USA) was used for all analyses. The level of significance was set at p < 0.05.

## Results

### Effects of R. junceus scorpion venom on cell viability in cancer and non-cancerous cell lines

**Figure 1.**
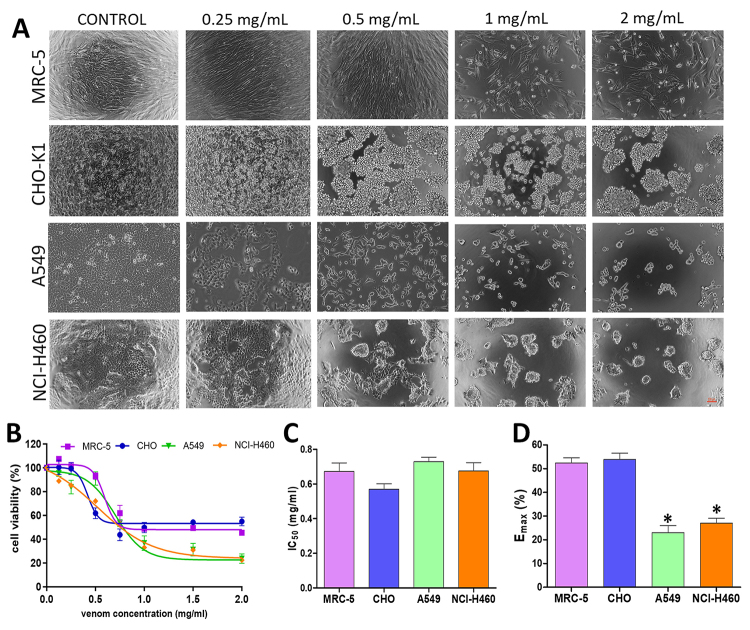
(A) Morphological effects in cancerous (A549, NCI-H460) and non-cancerous (MRC-5, CHO-K1) cells treated with R. junceus scorpion venom. (B) Cells were seeded in 96-well plates and treated for 72 h with increasing concentrations from 0.062 to 2 mg/mL. Dose-response curves were determined by MTT assay. The culture media was used as negative control. Results are expressed as the percentage of control. (C) IC_50_ values obtained from dose-response curves fitted to the Hill equation. (D) Maximum cell viability inhibition was obtained from the fit to the Hill equation (E_max_) in all cell lines at the highest scorpion venom concentration. *p < 0.05 compared to non-cancerous cells from Kruskal-Wallis non-parametric test. The experiments were performed three times with five technical replicates. Data values are expressed as mean ± SE. Scale bar: 100 µm.

Cells incubated for 72 h with various doses of scorpion venom showed morphological changes, including dose-dependent increases in cell monolayer rupture, cellular debris, and detached cells. Moreover, treated cells appeared more rounded, with cell membrane blebbing and axon-like protrusions. These changes were evident in the cancerous vs. non-cancerous cells incubated with R. junceus venom, as shown in Figure 1 A.

To confirm that the observed changes were due to a decrease in cell viability, we evaluated the effects of various concentrations of scorpion venom using MTT assay. As seen in Figure 1 B , the cells incubated with scorpion venom displayed a concentration-dependent decrease in cell viability after 72 hours of treatment. There were no significant differences in apparent IC_50_ values among the cell lines tested, with observed values of 0.71 ± 0.04 mg/mL, 0.57 ± 0.09 mg/mL, 0.68 ± 0.14 mg/mL, and 0.67 ± 0.15 mg/mL for MRC-5, CHO-K1, A549, and NCH-H460, respectively (Figure 1 C ). The maximal effects (E_max_) at the highest venom concentration tested (2 mg/mL) reached 52.39 ± 5.91% and 53.86 ± 7.52% cell viability for non-cancerous MRC-5 and CHO-K1 cells, compared to 23.02 ± 7.25% and 27.03 ± 5.15% for A549 and NCI-H460 cancer cells, indicating that R junceus venom displayed a higher potency against the cancer cell lines (Figure 1 D).

### Effects of R. junceus scorpion venom on cell cycle arrest in non-cancerous and cancerous cells 

**Figure 2.**
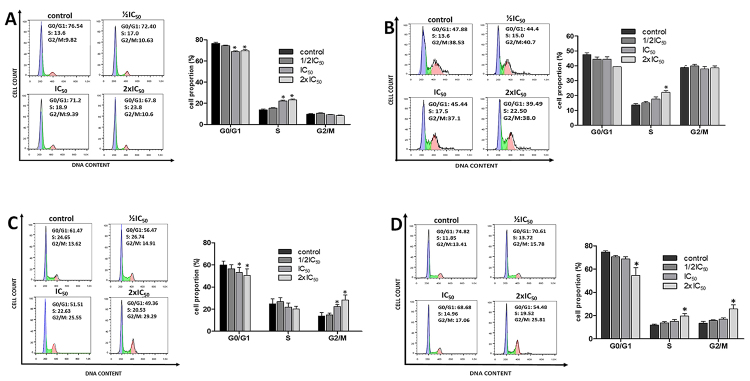
Effect of R. junceus scorpion venom on the cell cycle in non-cancerous and cancerous cells. **(A)** Representative histograms of the cell cycle in the MRC-5 cell line, after scorpion venom treatment for 48 h. Bar graph summary representing the percentage of cells in G0/G1, S, and G2/M in MRC-5 cells. **(B)** Representative histograms of the cell cycle in the CHO-K1 cell line, after scorpion venom treatment for 48 h. Bar graph summary representing the percentage of cells in G0/G1, S, and G2/M in CHO-K1cells. **(C)** Representative histograms of the cell cycle in the A549 cell line, after scorpion venom treatment for 48 h. Bar graph summary representing the percentage of cells in G0/G1, S, and G2/M in A549 cells. **(D)** Representative histograms of the cell cycle in the NCI-H460 cell line, after scorpion venom treatment for 48 h. Bar graph summary representing the percentage of cells in G0/G1, S, and G2/M in NCI-H460 cells. Cells (5 × 10^5^) were plated in 60 mm dishes and treated with ½IC_50_, IC_50,_ or 2xIC_50_ venom for 48 h. The cells were stained with propidium iodide, and the cell cycle was analyzed by flow cytometry. Each bar represents mean ± SE (n = 6). *p < 0.05 compared to control (the culture media in absence of scorpion venom) from Kruskal-Wallis non-parametric test.

To study the effects of R. junceus venom on the cell cycle, cells were incubated for 48 h (to minimize the confounding effects of primary cell death, especially at the highest doses) with the ½IC_50_, IC_50_, and 2xIC_50_ concentrations for each cell line, as previously established and shown in Figure 1. Flow cytometry cell cycle analysis using propidium iodide DNA staining was then performed. As expected, scorpion venom treatment modified the cell cycle distribution in a dose-dependent manner in all cell lines tested. Besides, there were differences in the cell cycle distribution between non-cancerous and cancerous cell lines (Figure 2).

The cell cycle profiles of non-cancerous MRC-5 and CHO-K1 cells after scorpion venom treatment are shown in Figures 2 A and 2B. Incubation of non-cancerous cells with scorpion venom increased significantly the percentage of cells in the S phase. In MRC-5 cells, the proportion of cells in the S phase increased from 13.6% ± 2.22% to 23.8% ± 2.32% (1.42 mg/mL venom concentration), with a concomitant decrease of cells in the G0/G1 phase (Figure 2 A ). Similarly, the percentage of CHO-K1 cells in the S phase increased significantly, from 13.62% ± 2.41% to 22.12% ± 2.8% (1.14 mg/mL venom concentration) (Figure 2 B ).

In contrast, both NSCLC cell lines showed a significant accumulation of cells in the G2/M phase after incubation with 1.36 mg/mL of scorpion venom, from 13.62 ± 3.3% to 29.29 ± 4.54% for A549 and from 13.41 ± 4.22% to 25.81 ± 9.03% for NCIH460 cells (Figures 2 C  and 2D).

### Detection of apoptotic cell death by flow cytometry in cancer cells

**Figure 3.**
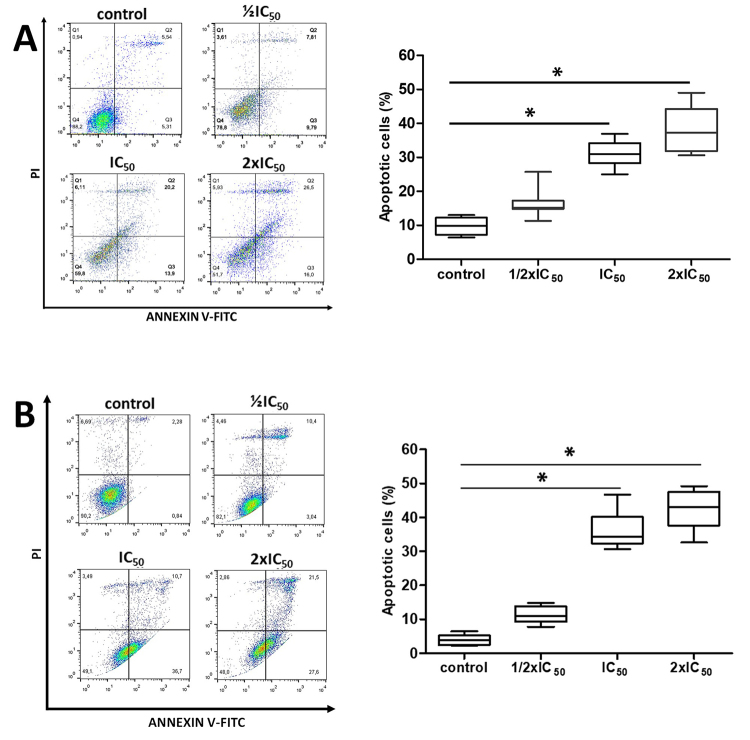
Analysis of apoptotic cell death in NSCLC A549 and NCI-H460 cells treated with R. junceus scorpion venom. Cells (5 × 10^5^) were plated in 60 mm dishes and treated with ½IC_50_, IC_50,_ or 2xIC_50_ venom for 48 h. Cells were stained with FITC-conjugated Annexin V and PI for flow cytometric analysis. **(A)** Representative scatter plot of PI (y-axis) and Annexin-V (x-axis) as measurements of apoptotic cell death. **(B)** Bar graph of the percentage of total apoptosis determined by flow cytometry (n = 6). *p < 0.05 compared to the control (the culture media in absence of scorpion venom) from the ANOVA test. Cells in the lower right (Annexin V^+^/PI^-^) represent early apoptosis and the upper right (Annexin V^+^/PI^+^) represent late apoptosis.

To investigate the type of cell death caused by R. junceus venom treatment, annexin V-FITC, and PI double-stained A549 and NCI-H460 cancer cells were analyzed with flow cytometry. As shown in Figure 3, incubation with scorpion venom triggered a significant increase in apoptosis in A549 cells, from 9.75 ± 2.50% in the control group to 31.0 ± 4.3% and 38.9 ± 2.4% for 0.68 mg/mL and 1.36 mg/mL respectively (Figure 3 A ).

Similarly, in NCI-H460 cells, the percentage of apoptotic cell death increased from 3.92 ± 1.56% in the control group to 36.1 ± 5.71% and 42.36 ± 6.09% for 0.67 mg/mL and 1.34 mg/mL, respectively (Figure 3 B ).

### Cell synchronization in A549 cancer cells

**Figure 4.**
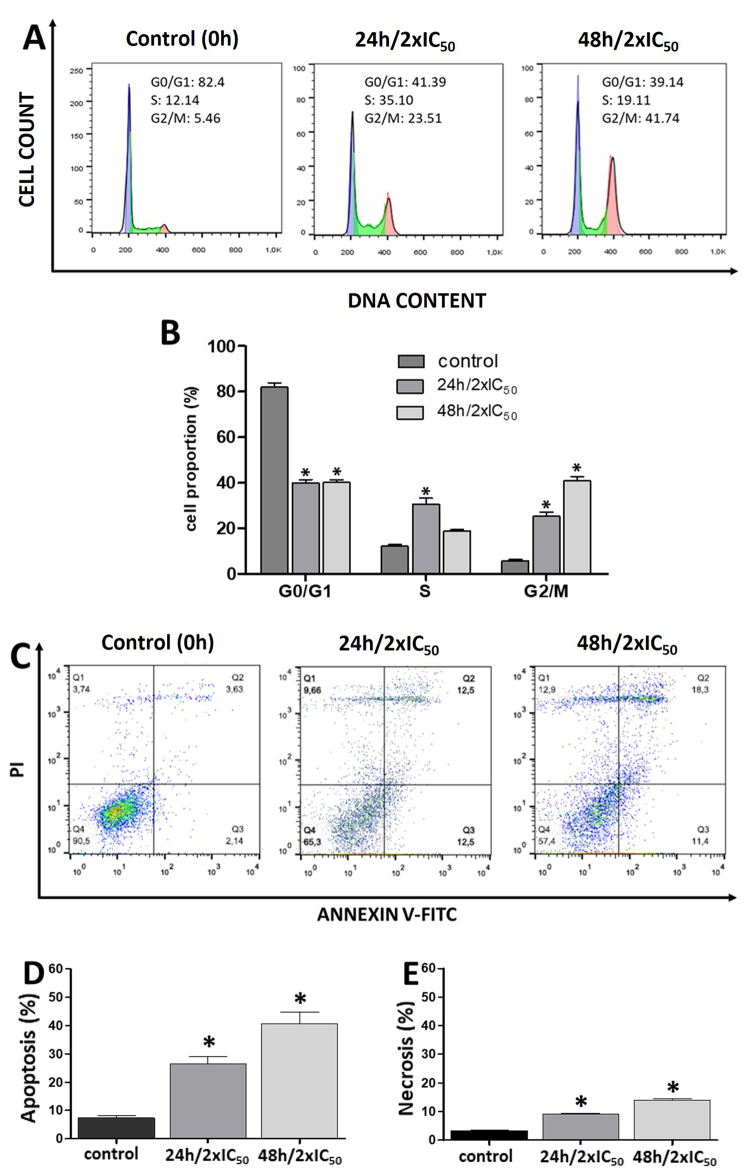
Analysis of cell cycle and apoptosis in serum-starved A549 cells treated with R. junceus scorpion venom (2xIC_50_) for 24 h, and 48 h after 10% FBS release. **(A)** Representative histograms of the cell cycle in A549 cells, after scorpion venom treatment. **(B)** Bar graph summary representing the percentage of cells in G0/G1, S, and G2/M. Bars represent an average of six measurements. **(C)** Representative scatter plot of PI (y-axis) and Annexin-V (x-axis) as measurements of apoptotic cell death. **(D)** Bar graph of the percentage of total apoptosis determined by flow cytometry (n = 6). **(E)** Bar graph of the percentage of necrosis determined by flow cytometry (n = 6). *p < 0.05 compared to control (the culture media in absence of scorpion venom) from the ANOVA test. Cells in the lower right (Annexin V^+^/PI^-^) represent early apoptosis and the upper right (Annexin V^+^/PI^+^) represent late apoptosis.

Next, we decided to corroborate the relationship between cell cycle arrest and apoptotic cell death after scorpion venom treatment. To this end, A549 cells were synchronized in G0/G1 phase by serum starvation for 72 h then incubated with scorpion venom (1.36 mg/mL) and 10% FBS for 24 or 48 hours. The proportion of cells in each cell cycle phase and the type of cell death was determined by flow cytometry (Figure 4); serum was reintroduced to allow the cell to re-enter the cell cycle.

Histograms of PI incorporation show a time-dependent increase in the proportion of cells arrested in the G2/M phase after scorpion venom treatment, from 5.80 ±1.03% (0 hours) to 25.27±3.85% at 24 h and 40.87 ± 3.31% after 48 h of incubation (Figure 4 A  and 4B). Similarly, the proportion of cells undergoing apoptosis showed a significant increase, with a concomitant increase in necrotic cell death (Figures 4 C  -4E).

### Western blot protein expression analysis after R. junceus scorpion venom treatment 

**Figure 5.**
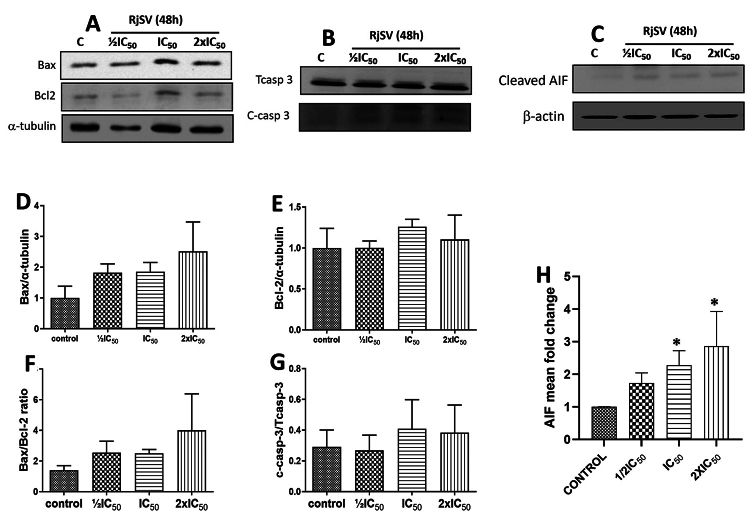
Relative protein expression of target proteins in A549 cells following R. junceus scorpion venom treatment at ½IC_50_, IC_50_, 2xIC_50_ for 48 h. Total cell lysates were obtained and then subjected to western blot analysis to measure the expression levels of proteins. **(A)** Western blot of apoptosis-related proteins Bax and Bcl-2. **(B)** Western blot of apoptosis-related protein caspase 3. **(C)** Western blot of cleaved-AIF protein. **(D)** Bar graph from Bax. **(E)** Bar graph from Bcl-2. **(F)** Bar graph from Bax/Bcl-2 ratio. **(G)** Bar graph from cleaved caspase 3/total caspase 3 ratio. Each bar represents mean ± SE (n = 3). **(H)** Bar graph of mean fold change from cleaved AIF protein. The mean fold change was plotted for each sample on a bar graph. *p < 0.05 compared to the control (the culture media in absence of scorpion venom) from Kruskal-Wallis non-parametric test. Bax and Bcl-2 signals were individually normalized to (-tubulin. AIF signal was normalized to β-Actin.

To determine the apoptotic pathway involved in the effect of the scorpion venom, the relative expression levels of apoptosis-related proteins in A549 were determined by Western blot. Treatment of A549 cancer cells with the scorpion venom does not induce significant changes in Bax or Bcl-2 expression levels as shown in Figure 5 (Figures 5 A , 5D, 5E).

Accordingly, the ratio of Bax/Bcl-2 proteins was similar in untreated and scorpion venom-treated cells (Figure 5 F ). Furthermore, cleaved caspase-3 levels in cells treated with venom did not differ significantly from those in untreated cells (Figures 5 B  and 5G). In contrast, the analysis of AIF expression levels revealed a dose-dependent increase in the AIF-cleaved form (see Figures 5 C and 5H, p < 0.05), while the total level of AIF protein remained unchanged (not shown).

**Figure 6.**
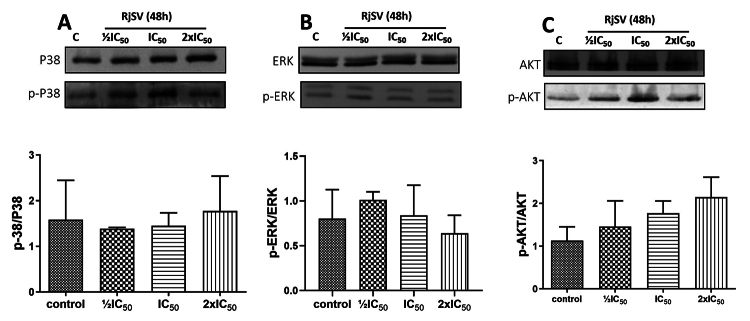
Relative protein expression of cell signaling-related proteins in A549 cells following R. junceus scorpion venom treatment at ½IC_50_, IC_50_, 2xIC_50_ for 48 h. **(A)** Western blot and bar graph from phosphorylated p38/total p38 ratio. **(B)** Western blot and bar graph from phosphorylated p42/44/total p42/44 ratio. **(C)** Western blot and Bar graph from phosphorylated pAKT/total AKT ratio. Each bar represents mean ± SE (n = 3). *p < 0.05 compared to control (the culture media in absence of scorpion venom) from Kruskal-Wallis non-parametric test.

We next analyzed the cell signaling-associated proteins P38 MAPK, ERK, and AKT following scorpion venom treatment. The scorpion venom showed no effect on p-P38 MAPK or p-ERK expression (Figures 6 A  and 6B). Likewise, no changes were observed on p-AKT (Figure 6 C ).

### Combined R. junceus scorpion venom and etoposide treatment 

The above results suggest that the scorpion venom induced cell cycle arrest in G2/M phase in NSCLC; therefore, we hypothesized that the scorpion venom should potentiate the cytotoxic effect of other G2/M phase-arresting drugs. To examine this idea, we tested the combined effect of scorpion venom and etoposide, a well-known topoisomerase II inhibitor.

**Figure 7.**
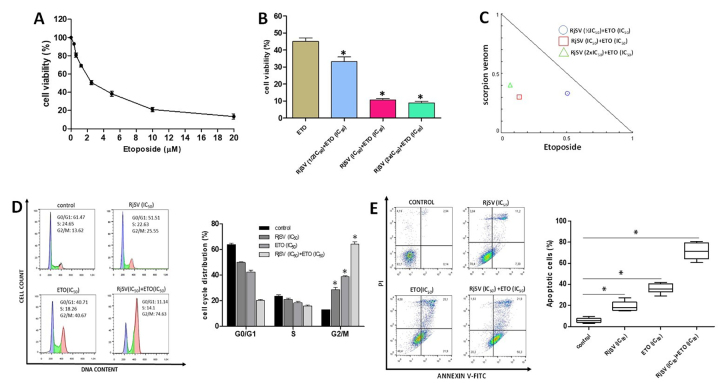
(A) Concentration-response curve from etoposide treatment in A549 cells. (B) Bar graph showing the cell viability determined by MTT assay in A549 cells at different concentrations of R. junceus venom (½IC_50_, IC_50_, 2xIC_50_) combined with etoposide (IC_50_). Untreated cells represent 100% cell viability. The culture medium was used as the negative control. *p < 0.05 respect to etoposide as a single treatment. The experiments were performed three times with five technical replicates. (C) Normalized isobologram of in vitro drug-to-drug interaction between R. junceus venom (RjSV) and etoposide (Eto) in A549 cancer cell line based on CompuSyn analysis from MTT data. (D) Representative cell cycle histogram and bar graph summary representing the percentage of cells in G0/G1, S, and G2/M after 48-h treatment. Bars represent an average of six measurements. (E) Representative scatter plot of PI (y-axis) and Annexin-V (x-axis) as measurements of apoptotic cell death and bar graph of the percentage of total apoptosis determined by flow cytometry (n = 6). *p < 0.05 compared to the control (the culture media in absence of scorpion venom) from the ANOVA test.

As shown in Figure 7 A , the A549 cells incubated with etoposide showed a dose-dependent decrease in cell viability after 72 h of treatment. The apparent IC_50_ value for this cell line was 2.53 ± 0.48 µM.

Thus, A549 cells were incubated with the etoposide (IC_50_) combined with various scorpion venom concentrations (½IC_50_, IC_50_, 2xIC_50_). Etoposide treatment reduced cell viability to 45.11 ± 4.91%. Meanwhile, the combined treatment (etoposide/scorpion venom) induced greater cytotoxicity than either drug alone (cell viability results of 33.16 ± 6.89%, 10.72 ± 1.91%, and 8.87 ± 2.45% respectively) (Figure 7 B ). The combination index analysis was used to confirm the type of interaction between the two drugs. From all three combined treatments, cytotoxicity ranged from 67-95%, reflecting a synergistic effect (C < 1), as illustrated in the isobologram in Figure 7 C .

The effects of single and combined treatments on cell cycle phases in the A549 line were also analyzed. The apparent IC_50_ value of each drug was used. Single treatments produced a significant accumulation of cells arrested at the G2/M phase for both scorpion venom (28.76 ± 3.40%) and etoposide (38.96 ± 1.95%) alone compared to the control (13.05 ± 1.94%) (p < 0.05). Moreover, the combined treatment provoked a significant increase of cells arrested at the G2/M phase (72.29 ± 7.34%) compared to control and single-treatment groups (p < 0.05) (Figure 7 D ). 

Next, the synergistic effect of scorpion venom and etoposide on cell death was studied. As previously shown, apoptotic cell death increased after incubation with scorpion venom (28.97 ± 4.89%) as well as etoposide (37.78 ± 6.4%) as single agents. However, when the two treatments were combined, the percentage of cells undergoing apoptosis was higher (71.88 ± 7.80%) (Figure 7 E ).

**Figure 8.**
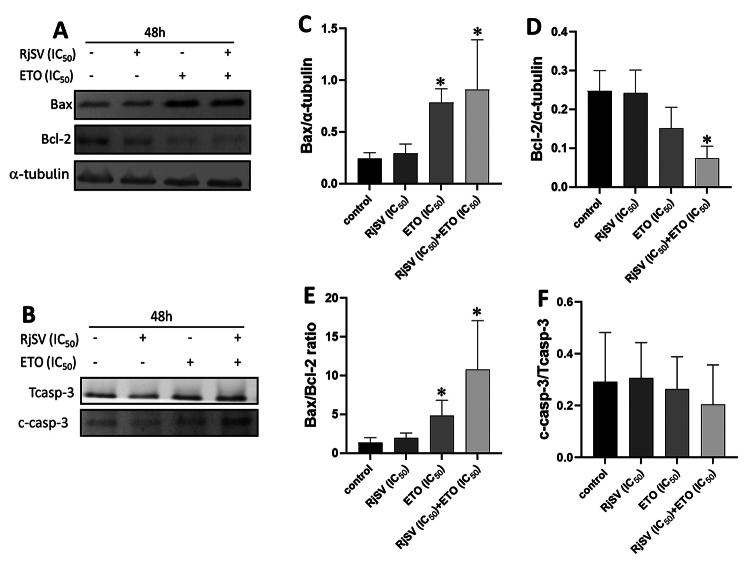
Relative protein expression of target proteins in A549 cells following R. junceus scorpion venom (IC_50_), etoposide (Eto, IC_50_), and combined treatment. **(A)** Western blot of apoptosis-related proteins Bax and Bcl-2. **(B)** Western blot of apoptosis-related protein caspase 3. **(C)** Bar graph from Bax. **(D)** Bar graph from Bcl-2. **(E)** Bar graph from Bax/Bcl-2 ratio. **(F)** Bar graph from cleaved caspase 3/total caspase 3 ratio. Each bar represents mean ± SE (n = 3). *p < 0.05 compared to control (the culture media in absence of scorpion venom) from Kruskal-Wallis non-parametric test. Bax and Bcl-2 signals were individually normalized to (-tubulin.

Finally, we determined the relative expression of apoptosis-related proteins in A549 cells from combined treatment. Figure 8 shows a marked increase in Bax expression levels in etoposide-treated A549 cells (p < 0.05), whereas the Bcl-2 proteins remained unchanged compared to the control (Figures 8 A, 8C, 8D).

Consequently, compared to control cells, the Bax/Bcl-2 ratio increased almost four times after etoposide treatment (IC_50_) (Figure 8 E ). Cleaved caspase-3 levels were similar to the control across all three treatments (Figure 8 B, 8F). Importantly, the venom/etoposide treatment increased Bax expression concomitant with decreased Bcl-2 expression (Figure 8 C , 8D), inducing a significant increase in the Bax/Bcl-2 ratio compared to the control and single-treatment groups (p < 0.05) (Figure 8 E ). Cleaved caspase-3 levels were not significantly affected by the combined treatments (Figure 8 F ).

### RP-HPLC separation and MTT assay in A549 cancer cell of isolated fractions

**Figure 9.**
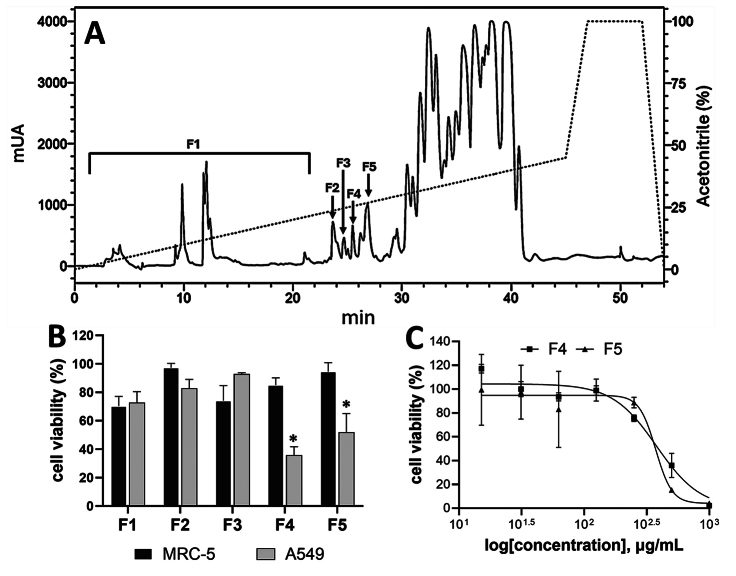
Reverse phase-HPLC chromatogram and MTT assay of Rhopalurus junceus scorpion venom fractions. **(A)** Reverse phase-HPLC chromatogram of R. junceus venom. Venom was injected into the C18-reverse phase column and run in HPLC. The run conditions were as follows: phase A - 0.1% TFA in HPLC grade water; phase B - 0.1% TFA in acetonitrile. Scorpion venom was eluted at 1 mL/min flow rate using a linear gradient of 0-45% of solvent B for 45 min. Fractions indicated with an arrow were collected individually from each run. **(B)** Evaluation of selected fractions at a unique concentration of 500 µg/mL, against MRC-5 and A549 cells. Cells were seeded in 96-well plates, treated for 72 h, and analyzed by MTT assay. **(C)** Dose-response curves of cells seeded in 96-well plates, treated for 72 h with increasing concentrations (15.1, 31.25, 62.5, 125, 250, 500, 1000 µg/mL) of fractions F4 and F5 and determined by MTT assay. IC_50_ values were obtained from dose-response curves fitted to the Hill equation. The culture medium was used as the negative control. Results were expressed as the percentage of control. *p < 0.05 compared to non-cancerous cells from Kruskal-Wallis non-parametric test. The experiments were performed three times with three technical replicates. Data values were expressed as mean ± SE.

We utilized high-performance liquid chromatography with a C18 reverse-phase column to pinpoint the soluble venom components affecting cell viability. The chromatographic profile, depicted in Figure 9, indicates that the crude venom contains 23 significant peaks that eluted between 3 and 42 minutes. The peptides eluting between 3-28 min retention times were separated into five fractions (Figure 9 A ) and the impact of each fraction on cell viability was studied in the lung cancer cell line A549 and the non-cancerous MRC-5 cells.

Fractions F4 and F5 at 0.5 mg/mL concentration reduced cell viability in the A549 cancer cell line (p < 0.05) but did not affect MRC-5 cells (Figure 9 B ). Nevertheless, fractions F1-3 did not affect cell viability in either cell line (Figure 9 B ). A549 cells incubation with increasing concentrations of the two most active fractions separately confirms that both fractions affect cell viability in a dose-dependent manner (Figure 9 C ). The IC_50_ values were 0.378±0.068 mg/mL and 0.373 ± 0.047 mg/mL for F4 and F5 respectively. 

## Discussion

In the present study, by comparing the effect of R. junceus scorpion venom against non-small cell lung cancer (A549, NCI-H460) and non-cancerous (MRC-5, CHO-K1) cell lines, we show that cancerous cells are more sensitive to the venom than non-cancerous cells in terms of monolayer integrity, morphology, and cytotoxicity. Dose-response curves indicate differences in the maximal effect reached despite similar IC_50_ values for all cell types (Figure 1), confirming a previous report demonstrating the selectivity of scorpion venom against lung cancer cells over the normal counterpart [13]. 

The Chinese hamster fibroblast cell line (CHO) is one of the most widely used to identify the toxicity of compounds [26, 27]. This cell line has several advantages, including a low chromosome number and large chromosome size, making it more sensitive to cytotoxic compounds in chromosome aberration tests compared to other mammalian cells [28]. Recently, tests using CHO cells have shown a strong correlation with in vivo toxicological mouse models and have been successful in predicting the initial dose for in vivo oral toxicity studies [29]. 

The MRC-5 fetal human fibroblast [30] is a widely used lung fibroblast cell line for studying the effects of cytotoxic drugs. MRC-5 has helped to determine safe concentration ranges for these drugs and their combination [31]. In lung cancer, the structural proteins known as cytokeratin serve as measurable markers due to their different expression compared to normal counterparts [32, 33]. These proteins are present in both fetal and adult lung tissues to the same extent [32]. 

Another commonly used cell line for cytotoxicity assays is human adult foreskin fibroblasts [34, 35]. According to gene expression analysis [36], MRC-5 and foreskin fibroblasts showed similar expression of most genes, with only a few being specific to each cell line [36]. This evidence suggests that there are no significant differences compared to adult tissue. Thus, all the evidence confirms that the cell lines used in this study are suitable models for identifying the potential toxicity of compounds.

To date, various studies have revealed the cytotoxic effects of scorpion venoms against some cancer cell lines, including colorectal, breast, leukemia, and cervical cancers [9, 37, 38]. However, only a few have compared the cancer cell phenotype with its normal cell counterpart. In human lung cancer, Androctonus australis venom and its toxic fraction exerted a greater effect against NCI-H358 cancer cells than normal MRC-5 fibroblasts [39]. As observed in the experiments, R. junceus venom can arrest cancer and non-cancerous cell lines at different cell cycle stages (Figure 2). To our knowledge, this is the first report detecting cancerous and non-cancerous cell lines arrested at distinct phases.

Venoms from poisoning animals usually induce cancer cell cycle arrest by upregulating various CDK inhibitors such as p16, p21, and p27. For example, Buthus martensii Karsch venom treatment in cancer cells upregulates p27, inducing cell cycle arrest at the G0/G1 phase [40]. Meanwhile, Macrothele raveni venom activates p21, arresting the cells at G2/M in the human hepatocellular carcinoma cell line HepG2 [41]. In both cases, cell cycle arrest precedes apoptotic cell death. In R. junceus venom, a previous study demonstrated an increase in p53 mRNA levels in MDA-MB-231 cells [15] with a concomitant increase in p21 mRNA, which could explain the arrest at G2/M observed in the present study (Figure 2) and the apoptotic cell death induced (Figure 3). However, the elevated levels of arrest at the S phase in non-cancerous cells (Figure 2) suggest that this venom contains other active peptides targeting proteins involved in the S checkpoint, an idea that needs further study.

We also examined the type of cell death incurred in the NSCLC cell lines after incubation with R. junceus scorpion venom. The early increase of phosphatidylserine exposure on the outer plasma membrane in cells (Figure 3) [42] and the absence of Bax/Bcl-2 ratio upregulation (Figure 5) suggest a mitochondria-dependent apoptosis [43-45].

Classical p53-dependent apoptosis is directly linked to p53 activation and increased expression of the proapoptotic proteins Bax, p21, and others. However, our experiments did not reveal an upregulation of mitochondrial apoptosis-related proteins such as cleaved caspase-3. The A549 cell line is a wild-type p53 cell line that lacks the CDKN2A locus [46], which contains the ARF gene responsible for P14^ARF^ expression. This protein sequesters MDM2 in the nucleolus, preventing p53 degradation and promoting its activation [47, 48]. Thus, it is feasible that the absence of the P14^ARF^ protein in A549 cells reduces the functional activity of p53, resulting in a mildly resistant phenotype for p53-dependent apoptosis stimuli. The same phenotype should be expected for most NSCLCs where the CDKN2A locus is absent (approximately 85% of such cancers) [46, 49].

We also found that combined treatment (R. junceus venom plus etoposide) did not increase cleaved caspase-3 levels in A549 cells, which is congruent with the absence of p53 activation. This result is consistent with previous reports of cancer cells treated with low doses of etoposide (0.5 µM), inducing apoptosis without altering cleaved caspase-3, -9, or -8 levels [50]. Etoposide can cause either caspase-dependent or -independent apoptotic cell death depending on the dose, with caspase-independent apoptosis occurring at low doses [50, 51]. The caspase-independent apoptosis induced by etoposide is believed to be mediated by DNA damage response signaling, given the anti-topoisomerase II activity of this drug [22]. In addition, the mitochondria play a crucial role in regulating caspase-independent apoptotic cell death by activating certain calpains and cathepsins. This activation results in the translocation of Bax from the cytoplasm to the mitochondria. The presence of Bax and the cleaved Bid proteins on the mitochondrial membrane causes a loss of mitochondrial membrane potential, which leads to increased membrane permeability and the release of cleaved AIF (apoptosis-inducing factor) [52].

Therefore, the release of cleaved AIF results from mitochondrial membrane potential destabilization, leading to mitochondrial outer membrane permeabilization, loss of mitochondrial function, and ultimately causing DNA degradation during apoptotic cell death.

In this study, we found that scorpion venom increases the cleaved form of AIF, indicating the release of AIF from mitochondria to the cytosol. This result suggests that the scorpion venom induces apoptosis by promoting cell cycle arrest (Figure 3), a known mechanism upon AIF release [52-54]. As expected, for AIF-dependent apoptosis, no changes in the phosphorylation levels of P38 MAPK, ERK, or AKT were found upon venom treatment in the A549 cells (Figure 6). However, the precise mechanism that led to AIF release requires further study.

Our findings support a model in which scorpion venom causes caspase-independent apoptotic cell death, likely due to a p53-resistant phenotype and mitochondrial-dependent. In this situation, the drug etoposide would increase the venom's cytotoxic effect by impeding DNA repair. The combination of R. junceus venom and etoposide induces a significant decrease in the proportion of viable A549 cancer cells when compared to either individual treatment. This result suggests a synergistic effect, as illustrated in Figure 8. Additionally, there was a significant increase in apoptosis and cell cycle arrest at G2/M with the combined treatment (Figure 8), supporting the advantage of the combination therapy. 

Other natural products have been shown to heighten the effectiveness of etoposide [55]. For instance, some polyphenols disrupt the ATM-Chk1 pathway, inhibiting DNA damage checkpoints and repair pathways [56], while curcumin reduces glucose uptake and lactate production (Warburg effect) [57]. The combination of curcumin and etoposide promotes apoptosis in gastric cancer cells by deregulating the NF-κB and HIF-1 pathways [55]. Additionally, combining resveratrol with etoposide has been found to downregulate the expression of cyclin D1, cyclin D2, and cyclin E, inhibiting CDK2, CDK4, and CDK6 activities and expression while upregulating p21 expression [58, 59] and increasing apoptotic activities [55, 60]. Therefore, natural products show varying synergistic mechanisms to enhance the anticancer effects of etoposide.

 Cell viability studies were conducted using fractions of scorpion venom eluting between 3-28 minutes. According to prior research [61], these fractions typically contain low molecular weight peptides [62-64]; including ion channels-interacting peptides that recognize K+ channels, a type of ion channel with demonstrated roles in cancer [4]. Moreover, previous evidence suggests that most scorpion venom peptides with anticancer activity are found within this retention time frame [65, 66].

It is interesting to note that only F4 and F5 fractions showed a different effect on cell viability between A549, and MRC-5 cell lines when different soluble protein fractions were used. The reason for this difference is currently unclear, but previous studies have indicated that MRC-5 cells have lower expression of certain membrane proteins compared to A549 cells [67]. A comprehensive comparative proteomic analysis has previously shown that these cells express different membrane and non-membrane proteins, including some potassium channels [68]. Therefore, it is likely that the peptides in these fractions include some ion channel-interacting proteins, which may explain the in vitro cytotoxic effect of R. junceus scorpion venom described in this study.

## Conclusion

 Here we show that venom from R. junceus scorpion is more effective at reducing cell viability of non-small cell lung cancer (NSCLC) cells compared to the non-cancerous counterparts. The mechanism of action seems to involve AIF release from mitochondria, cell cycle arrest, and apoptosis in a caspase-independent manner. When the scorpion venom is combined with etoposide, a well-known chemotherapeutic agent, it enhances the effect of both treatments. Initial findings indicate that specific venom fractions are responsible for the observed effect. However, further experiments are necessary to identify the specific compounds targeting NSCLC cells and to understand their relationship with the cell cycle arrest and mitochondrial-dependent apoptosis induction caused by the scorpion venom treatment.

## Supplementary material

The following online material is available for this article:

Additional file 1. Raw data for Figure 5. Original membrane blots (left) and the resulting image (right) presented in the manuscript are shown. Signals enclosed in the dashed red rectangles were used for the final figures.

Additional file 2. Raw data for figure 6. Original membrane blots (left) and the resulting image (right) presented in the manuscript are shown. Signals enclosed in the dashed red rectangles were used for the final figures.

Additional file 3. Raw data for figure 8. Original membrane blots (left) and the resulting image (right) presented in the manuscript are shown. Signals enclosed in the dashed red rectangles were used for the final figures.

## References

[B1] Sung H, Ferlay J, Siegel RL, Laversanne M, Soerjomataram I, Jemal A, Bray F (2021). Global Cancer Statistics 2020: GLOBOCAN Estimates of Incidence
and Mortality Worldwide for 36 Cancers in 185 Countries. CA Cancer J Clin.

[B2] Thai AA, Solomon BJ, Sequist LV, Gainor JF, Heist RS (2021). Lung cancer. Lancet.

[B3] Dutta S, Mahalanobish S, Saha S, Ghosh S, Sil P (2019). Natural products: An upcoming therapeutic approach to
cancer. Food Chem Toxicol.

[B4] Diaz-Garcia A, Varela D (2020). Voltage-Gated K(+)/Na(+) Channels and Scorpion Venom Toxins in
Cancer. Front Pharmacol.

[B5] Raposo C (2017). Scorpion and spider venoms in cancer treatment: state of the art,
challenges, and perspectives. J Clin Transl Res.

[B6] Al-Asmari AK, Islam M, Al-Zahrani AM (2016). In vitro analysis of the anticancer properties of scorpion venom
in colorectal and breast cancer cell lines. Oncol Lett.

[B7] Al-Asmari AK, Riyasdeen A, Abbasmanthiri R, Arshaduddin M, Al-Harthi FA (2016). Scorpion (Androctonus bicolor) venom exhibits cytotoxicity and
induces cell cycle arrest and apoptosis in breast and colorectal cancer cell
lines. Indian J Pharmacol.

[B8] Al-Asmari AK, Riyasdeen A, Islam M (2018). Scorpion Venom Causes Apoptosis by Increasing Reactive Oxygen
Species and Cell Cycle Arrest in MDA-MB-231 and HCT-8 Cancer Cell
Lines. J Evid Based Integr Med.

[B9] Al-Asmari AK, Riyasdeen A, Islam M (2018). Scorpion Venom Causes Upregulation of p53 and Downregulation of
Bcl-xL and BID Protein Expression by Modulating Signaling Proteins Erk(1/2)
and STAT3, and DNA Damage in Breast and Colorectal Cancer Cell
Lines. Integr Cancer Ther.

[B10] Zargan J, Sajad M, Umar S, Naime M, Ali S, Khan HA (2011). Scorpion (Androctonus crassicauda) venom limits growth of
transformed cells (SH-SY5Y and MCF-7) by cytotoxicity and cell cycle
arrest. Exp Mol Pathol.

[B11] Zargan J, Umar S, Sajad M, Naime M, Ali S, Khan HA (2011). Scorpion venom (Odontobuthus doriae) induces apoptosis by
depolarization of mitochondria and reduces S-phase population in human
breast cancer cells (MCF-7). Toxicol In Vitro.

[B12] Ahmadi S, Knerr JM, Argemi L, Bordon KCF, Pucca MB, Cerni FA, Arantes EC, Caliskan F, Laustsen AH (2020). Scorpion Venom: Detriments and Benefits. Biomedicines.

[B13] Diaz-Garcia A, Morier-Diaz L, Frion-Herrera Y, Rodriguez-Sanchez H, Caballero-Lorenzo Y, Mendoza-Llanes D, Riquenes-Garlobo Y, Fraga-Castro JA (2013). In vitro anticancer effect of venom from Cuban scorpion
Rhopalurus junceus against a panel of human cancer cell
lines. J Venom Res.

[B14] Díaz-García A, Ruiz-Fuentes JL, Frión-Herrera Y, Yglesias-Rivera A, Riquenez Garlobo Y, Rodríguez Sánchez H, Rodríguez Aurrecochea JC, López Fuentes LX (2019). Rhopalurus junceus scorpion venom induces antitumor effect in
vitro and in vivo against a murine mammary adenocarcinoma
model. Iran J Basic Med Sci.

[B15] Diaz-Garcia A, Ruiz-Fuentes JL, Rodriguez-Sanchez H, Fraga Castro JA (2017). Rhopalurus junceus scorpion venom induces apoptosis in the triple
negative human breast cancer cell line MDA-MB-231. J Venom Res.

[B16] Matthews HK, Bertoli C, de Bruin RAM (2022). Cell cycle control in cancer. Nat Rev Mol Cell Biol.

[B17] Liu W, Jin W, Zhu S, Chen Y, Liu B (2022). Targeting regulated cell death (RCD) with small-molecule
compounds in cancer therapy: A revisited review of apoptosis,
autophagy-dependent cell death and necroptosis. Drug Discov Today.

[B18] Sun Y, Liu Y, Ma X, Hu H (2021). The Influence of Cell Cycle Regulation on
Chemotherapy. Int J Mol Sci.

[B19] Ohneseit PA, Prager D, Kehlbach R, Rodemann HP (2005). Cell cycle effects of topotecan alone and in combination with
irradiation. Radiother Oncol.

[B20] Kciuk M, Gielecinska A, Mujwar S, Kolat D, Kaluzinska-Kolat Z, Celik I, Kontek R (2023). Doxorubicin-An Agent with Multiple Mechanisms of Anticancer
Activity. Cells.

[B21] Kothari A, Hittelman WN, Chambers TC (2016). Cell Cycle-Dependent Mechanisms Underlie Vincristine-Induced
Death of Primary Acute Lymphoblastic Leukemia Cells. Cancer Res.

[B22] Baldwin EL, Osheroff N (2005). Etoposide, topoisomerase II and cancer. Curr Med Chem Anticancer Agents.

[B23] Meresse P, Dechaux E, Monneret C, Bertounesque E (2004). Etoposide: discovery and medicinal chemistry. Curr Med Chem.

[B24] Mosmann T (1983). Rapid colorimetric assay for cellular growth and survival:
application to proliferation and cytotoxicity assays. J Immunol Methods.

[B25] Chou TC (2006). Theoretical basis, experimental design, and computerized
simulation of synergism and antagonism in drug combination
studies. Pharmacol Rev.

[B26] Clare G (2012). The in vitro mammalian chromosome aberration test. Methods Mol Biol.

[B27] Miller B, Albertini S, Locher F, Thybaud V, Lorge E (1997). Comparative evaluation of the in vitro micronucleus test and the
in vitro chromosome aberration test: industrial experience. Mutat Res.

[B28] Mark HF, Naram R, Pham T, Shah K, Cousens LP, Wiersch C, Airall E, Samy M, Zolnierz K, Mark R (1994). A practical cytogenetic protocol for in vitro cytotoxicity and
genotoxicity testing. Ann Clin Lab Sci.

[B29] Lin YE, Lin MH, Yeh TY, Lai YS, Lu KH, Huang HS, Peng FC, Liu SH, Sheen LY (2022). Genotoxicity and 28-day repeated dose oral toxicity study of
garlic essential oil in mice. J Tradit Complement Med.

[B30] Jacobs JP, Jones CM, Baille JP (1970). Characteristics of a human diploid cell designated
MRC-5. Nature.

[B31] Duarte D, Nunes M, Ricardo S, Vale N (2022). Combination of Antimalarial and CNS Drugs with Antineoplastic
Agents in MCF-7 Breast and HT-29 Colon Cancer Cells: Biosafety Evaluation
and Mechanism of Action. Biomolecules.

[B32] Broers JL, de Leij L, Rot MK, ter Haar A, Lane EB, Leigh IM, Wagenaar SS, Vooijs GP, Ramaekers FC (1989). Expression of intermediate filament proteins in fetal and adult
human lung tissues. Differentiation.

[B33] Buccheri G, Ferrigno D (2001). Lung tumor markers of cytokeratin origin: an
overview. Lung Cancer.

[B34] Al-Faifi ZI, Masrahi YS, Aly MS, Al-Turki TA, Dardeer T (2017). Evaluation of Cytotoxic and Genotoxic Effects of Euphorbia
Triaculeata Forssk. Extract. Asian Pac J Cancer Prev.

[B35] Majoumouo MS, Tincho MB, Kouipou Toghueo RM, Morris T, Hiss DC, Boyom FF, Mandal C (2020). Cytotoxicity Potential of Endophytic Fungi Extracts from
Terminalia catappa against Human Cervical Cancer Cells. J Toxicol.

[B36] Marthandan S, Priebe S, Baumgart M, Groth M, Cellerino A, Guthke R, Hemmerich P, Diekmann S (2015). Similarities in Gene Expression Profiles during In Vitro Aging of
Primary Human Embryonic Lung and Foreskin Fibroblasts. Biomed Res Int.

[B37] Bernardes-Oliveira E, Farias KJS, Gomes DL, de Araujo JMG, da Silva WD, Rocha HAO, Donadi EA, Fernandes-Pedrosa MF, Crispim JCO (2019). Tityus serrulatus Scorpion Venom Induces Apoptosis in Cervical
Cancer Cell Lines. Evid Based Complement Alternat Med.

[B38] Das Gupta S, Debnath A, Saha A, Giri B, Tripathi G, Vedasiromoni J, Gomes A, Gomes A (2007). Indian black scorpion (Heterometrus bengalensis Koch) venom
induced antiproliferative and apoptogenic activity against human leukemic
cell lines U937 and K562. Leuk Res.

[B39] Béchohra L, Laraba-Djebari F, Hammoudi-Triki D (2016). Cytotoxic activity of Androctonus australis hector venom and its
toxic fractions on human lung cancer cell line. J Venom Anim Toxins incl Trop Dis.

[B40] Gao F, Li H, Chen YD, Yu XN, Wang R, Chen XL (2009). Upregulation of PTEN involved in scorpion venom-induced apoptosis
in a lymphoma cell line. Leuk Lymphoma.

[B41] Gao L, Shen JB, Sun J, Shan BE (2007). Effect of the venom of the spider Macrothele raveni on the
expression of p21 gene in HepG2 cells. Sheng Li Xue Bao.

[B42] Nagata S, Suzuki J, Segawa K, Fujii T (2016). Exposure of phosphatidylserine on the cell
surface. Cell Death Differ.

[B43] Karmakar S, Banik NL, Ray SK (2007). Curcumin suppressed anti-apoptotic signals and activated cysteine
proteases for apoptosis in human malignant glioblastoma U87MG
cells. Neurochem Res.

[B44] Raisova M, Hossini AM, Eberle J, Riebeling C, Wieder T, Sturm I, Daniel PT, Orfanos CE, Geilen CC (2001). The Bax/Bcl-2 ratio determines the susceptibility of human
melanoma cells to CD95/Fas-mediated apoptosis. J Invest Dermatol.

[B45] Zhu L, Han MB, Gao Y, Wang H, Dai L, Wen Y, Na LX (2015). Curcumin triggers apoptosis via upregulation of Bax/Bcl-2 ratio
and caspase activation in SW872 human adipocytes. Mol Med Rep.

[B46] Nicholson SA, Okby NT, Khan MA, Welsh JA, McMenamin MG, Travis WD, Jett JR, Tazelaar HD, Trastek V, Pairolero PC, Corn PG, Herman JG, Liotta LA, Caporaso NE, Harris CC (2001). Alterations of p14ARF, p53, and p73 genes involved in the
E2F-1-mediated apoptotic pathways in non-small cell lung
carcinoma. Cancer Res.

[B47] Cilluffo D, Barra V, Di Leonardo A (2020). P14(ARF): The Absence that Makes the Difference. Genes (Basel).

[B48] Inoue K, Fry EA (2019). Aberrant Expression of p14(ARF) in Human Cancers: A New
Biomarker?. Tumor Microenviron.

[B49] Fontana R, Ranieri M, La Mantia G, Vivo M (2019). Dual Role of the Alternative Reading Frame ARF Protein in
Cancer. Biomolecules.

[B50] Bruni E, Reichle A, Scimeca M, Bonanno E, Ghibelli L (2018). Lowering Etoposide Doses Shifts Cell Demise From
Caspase-Dependent to Differentiation and Caspase-3-Independent Apoptosis via
DNA Damage Response, Inducing AML Culture Extinction. Front Pharmacol.

[B51] Zhang SH, Huang Q (2013). Etoposide induces apoptosis via the mitochondrial- and
caspase-dependent pathways and in non-cancer stem cells in Panc-1 pancreatic
cancer cells. Oncol Rep.

[B52] Bhadra K (2022). A Mini Review on Molecules Inducing Caspase-Independent Cell
Death: A New Route to Cancer Therapy. Molecules.

[B53] Sevrioukova IF (2011). Apoptosis-inducing factor: structure, function, and redox
regulation. Antioxid Redox Signal.

[B54] Zong L, Liang Z (2023). Apoptosis-inducing factor: a mitochondrial protein associated
with metabolic diseases-a narrative review. Cardiovasc Diagn Ther.

[B55] Kluska M, Wozniak K (2021). Natural Polyphenols as Modulators of Etoposide Anti-Cancer
Activity. Int J Mol Sci.

[B56] Ahmed S, Alam W, Aschner M, Alsharif KF, Albrakati A, Saso L, Khan H (2022). Natural products targeting the ATR-CHK1 signaling pathway in
cancer therapy. Biomed Pharmacother.

[B57] Siddiqui FA, Prakasam G, Chattopadhyay S, Rehman AU, Padder RA, Ansari MA, Irshad R, Mangalhara K, Bamezai RNK, Husain M, Ali SM, Iqbal MA (2018). Curcumin decreases Warburg effect in cancer cells by
down-regulating pyruvate kinase M2 via mTOR-HIF1alpha
inhibition. Sci Rep.

[B58] Singh SK, Banerjee S, Acosta EP, Lillard JW, Singh R (2017). Resveratrol induces cell cycle arrest and apoptosis with
docetaxel in prostate cancer cells via a p53/ p21WAF1/CIP1 and p27KIP1
pathway. Oncotarget.

[B59] Yuan L, Zhang Y, Xia J, Liu B, Zhang Q, Liu J, Luo L, Peng Z, Song Z, Zhu R (2014). Resveratrol induces cell cycle arrest via a p53-independent
pathway in A549 cells. Mol Med Rep.

[B60] Heiduschka G, Bigenzahn J, Brunner M, Thurnher D (2014). Resveratrol synergistically enhances the effect of etoposide in
HNSCC cell lines. Acta Otolaryngol.

[B61] Rodriguez-Ravelo R, Coronas FI, Zamudio FZ, Gonzalez-Morales L, Lopez GE, Urquiola AR, Possani LD (2013). The Cuban scorpion Rhopalurus junceus (Scorpiones, Buthidae):
component variations in venom samples collected in different geographical
areas. J Venom Anim Toxins incl Trop Dis.

[B62] Dai L, Yasuda A, Naoki H, Corzo G, Andriantsiferana M, Nakajima T (2001). IsCT, a novel cytotoxic linear peptide from scorpion
Opisthacanthus madagascariensis. Biochem Biophys Res Commun.

[B63] Gurrola GB, Hernandez-Lopez RA, Rodriguez de la Vega RC, Varga Z, Batista CV, Salas-Castillo SP, Panyi G, del Rio-Portilla F, Possani LD (2012). Structure, function, and chemical synthesis of Vaejovis mexicanus
peptide 24: a novel potent blocker of Kv1.3 potassium channels of human T
lymphocytes. Biochemistry.

[B64] Vandendriessche T, Kopljar I, Jenkins DP, Diego-Garcia E, Abdel-Mottaleb Y, Vermassen E, Clynen E, Schoofs L, Wulff H, Snyders D, Tytgat J (2012). Purification, molecular cloning and functional characterization
of HelaTx1 (Heterometrus laoticus): the first member of a new kappa-KTX
subfamily. Biochem Pharmacol.

[B65] Aissaoui D, Mlayah-Bellalouna S, Jebali J, Abdelkafi-Koubaa Z, Souid S, Moslah W, Othman H, Luis J, ElAyeb M, Marrakchi N, Essafi-Benkhadir K, Srairi-Abid N (2018). Functional role of Kv1.1 and Kv1.3 channels in the neoplastic
progression steps of three cancer cell lines, elucidated by scorpion
peptides. Int J Biol Macromol.

[B66] Schickling BM, England SK, Aykin-Burns N, Norian LA, Leslie KK, Frieden-Korovkina VP (2015). BKCa channel inhibitor modulates the tumorigenic ability of
hormone-independent breast cancer cells via the Wnt pathway. Oncol Rep.

[B67] Jang SH, Ryu PD, Lee SY (2011). Dendrotoxin-kappa suppresses tumor growth induced by human lung
adenocarcinoma A549 cells in nude mice. J Vet Sci.

[B68] Rubporn A, Srisomsap C, Subhasitanont P, Chokchaichamnankit D, Chiablaem K, Svasti J, Sangvanich P (2009). Comparative proteomic analysis of lung cancer cell line and lung
fibroblast cell line. Cancer Genomics Proteomics.

