# Supplementary material for: Rhopalurus junceus scorpion venom induces G2/M cell cycle arrest and apoptotic cell death in human non-small lung cancer cell lines
Source: J Venom Anim Toxins Incl Trop Dis. 2025 Feb 3;31:e20240035. doi: 10.1590/1678-9199-JVATITD-2024-0035 (PMC11792888; doi:10.1590/1678-9199-JVATITD-2024-0035)
Supplement: Additional file 2. [file 1678-9199-jvatitd-31-e20240035-s2.pdf]

J Venom Anim Toxins incl Trop Dis

https://doi.org/10.1590/1678-9199-JVATITD-2024-0035

Supplementary Material to "*Rhopalurus junceus* scorpion venom induces G2/M cell cycle arrest and apoptotic cell death in human non-small lung cancer cell lines"

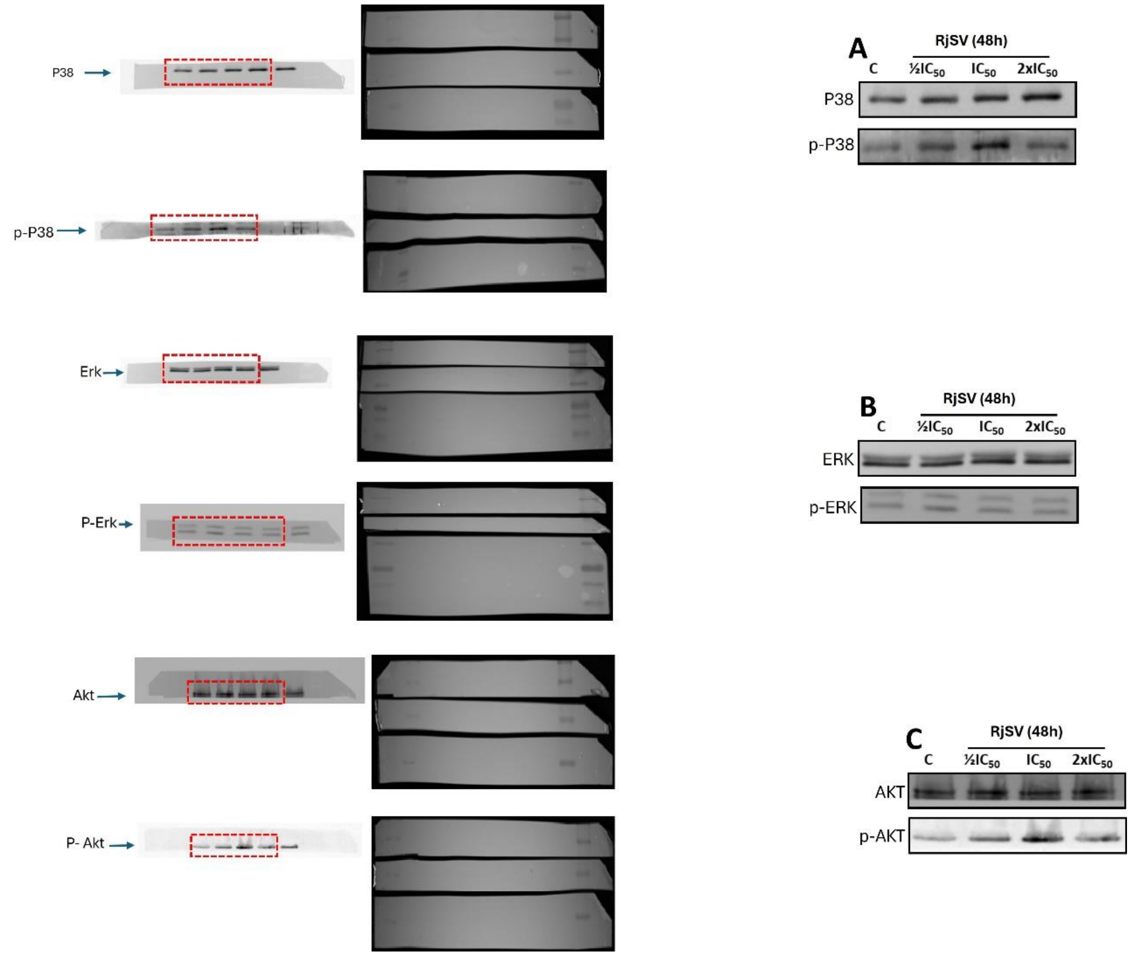

Additional file 2. Raw data for figure 6. Original membrane blots (left) and the resulting image (right) presented in the manuscript are shown. Signals enclosed in the dashed red rectangles were used for the final figures.
